# SURF: identifying and allocating resources during Out-of-Hospital Cardiac Arrest

**DOI:** 10.1186/s12911-020-01334-4

**Published:** 2020-12-30

**Authors:** Gaurav Rao, Salimur Choudhury, Pawan Lingras, David Savage, Vijay Mago

**Affiliations:** 1 Department of Mathematics and Computing Science, Saint Mary’s University, Halifax, CA USA; 2 Department of Computer Science, Lakehead University, Thunder Bay, CA USA; 3 Northern Ontario School of Medicine, Thunder Bay, CA USA

**Keywords:** Out-of-Hospital Cardiac Arrest (OHCA), Resource management, Optimization and allocation, Integer linear programming (ILP), Internet of things (IoT)

## Abstract

**Background:**

When an Out-of-Hospital Cardiac Arrest (OHCA) incident is reported to emergency services, the 911 agent dispatches Emergency Medical Services to the location and activates responder network system (RNS), if the option is available. The RNS notifies all the registered users in the vicinity of the cardiac arrest patient by sending alerts to their mobile devices, which contains the location of the emergency. The main objective of this research is to find the best match between the user who could support the OHCA patient.

**Methods:**

For performing matching among the user and the AEDs, we used Bipartite Matching and Integer Linear Programming. However, these approaches take a longer processing time; therefore, a new method Preprocessed Integer Linear Programming is proposed that solves the problem faster than the other two techniques.

**Results:**

The average processing time for the experimentation data was   1850 s using Bipartite matching,   32 s using the Integer Linear Programming and  2 s when using the Preprocessed Integer Linear Programming method. The proposed algorithm performs matching among users and AEDs faster than the existing matching algorithm and thus allowing it to be used in the real world.

**Conclusion:**

This research proposes an efficient algorithm that will allow matching of users with AED in real-time during cardiac emergency. Implementation of this system can help in reducing the time to resuscitate the patient.

## Background

Sudden Cardiac Arrest (SCA) is a medical condition in which a person’s heart stops beating due to the failure of the heart’s electrical system [1]. During SCA, the patient’s survival chances reduce by 10% per minute [2–6]. The SCA patient should be immediately provided with Cardiopulmonary Resuscitation (CPR), along with an electrical shock using Automated External Defibrillator (AED). SCA is the cause of over 350,000 deaths per year in the U.S. and approximately 34,000 in Canada [7–10].

Early resuscitation helps in increasing the survival chances of cardiac arrest patients [11]. Studies showed an increase of up to 24% in the survival chances of the patients when bystanders provided resuscitation before the arrival of emergency services [3, 11, 12]. Therefore it is essential to reduce the time taken by either the emergency medical services or nearby users to reach the patient and provide resuscitation [4–6]. Studies also showed an increase in patient survivability when a nearby user applied an AED on the patient compared to no AED applied to the patient; this implies that an AED should be applied to the SCA patient at the earliest time possible [4–6].

Medical and emergency teams align with the point that early resuscitation is essential in saving a patient’s life. Therefore, emergency services (like 911) have started implementing the Responder network system (RNS). Currently, only a small number of emergency departments have deployed RNSs. As per literature, the PulsePoint Respond is the only RNS deployed in the U.S. and that too only by a limited number of emergency dispatchers [13]. Stockholm and the Swiss canton of Ticino have also deployed RNSs among other RNS deployed locations [14, 15]. Researchers had studied the impact of deploying RNS in these locations and found that after implementing RNS, more cardiac arrest patients survived when users who are notified via RNS arrived at the emergency location before the ambulance services arrived [11, 13, 14].

**Fig. 1:**
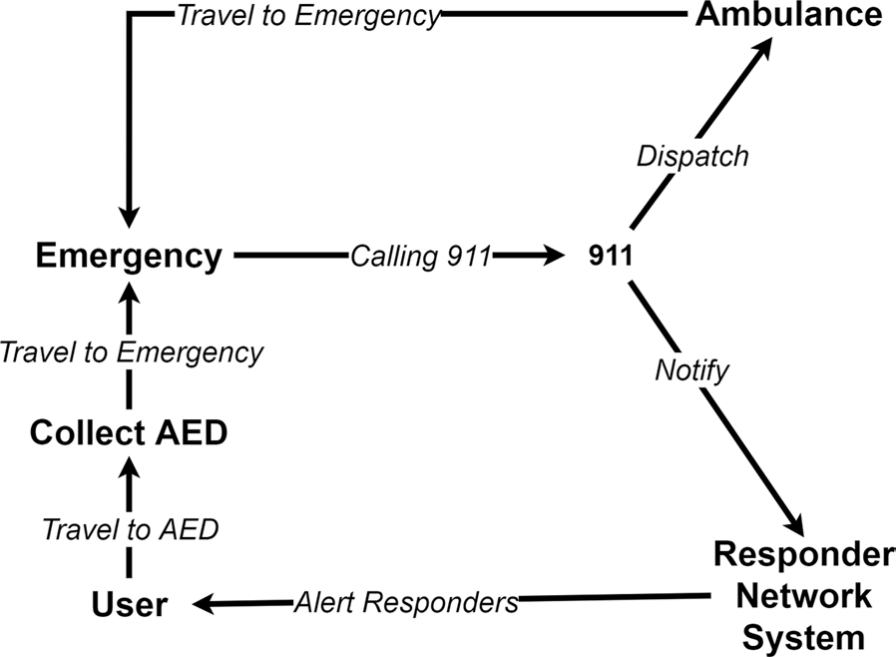
Emergency workflow during a cardiac arrest event

The RNS is linked with the emergency services, and it is activated by the emergency call attendant when a cardiac arrest is reported. The call attendee provides the location of the emergency when activating it. The RNS then identifies and notifies all the registered users that are within a specified radius of the emergency location. The notification contains the emergency location and is delivered either by an SMS or as a mobile application notification. The notified users then try to find a nearby AED either by using another mobile application or trying to recall any nearby AED location. Then they carry the AED to the emergency location. Figure 1, shows the workflow. However, the RNS has drawbacks and, if resolved, can increase the survival chances of the patients.

A major drawback in the current RNS implementation is that it notifies all the users in a given radius of the emergency location, which means if the population’s density is high (like New York, Beijing, Toronto), it could notify hundreds of users. Many notifications imply that precious human resources are over-utilized, and after a while, the users will not be taking notifications seriously. A study shows that the users may be less responsive if they could not help the patient in previous attempts [13]. Such situations can be like false RNS notifications; the user cannot locate the patient; the user arrives after the arrival of Emergency Medical Services (EMS), and other similar situations. Therefore, it is crucial to notify only a limited number of users that can arrive at the emergency before the arrival of emergency services.

When users receive an emergency notification, they need to find a nearby AED and carry it to the emergency. To find a nearby AED, the users rely either on their knowledge about AED locations or use mobile applications such as AED Quebec, Staying Alive, Pulse point Respond [16–18]. These applications are free and available on both the Apple App Store and Google Play Store [19, 20]. These mobile applications use the crowdsourcing method to collect the location and other information about the AEDs. This data collection technique is not reliable, as the data is not cross-validated. After receiving the notification, the user opens an AED finder mobile application and then tries to find an AED on its way to the emergency. This process of finding an AED may take away a few crucial minutes. For saving the time to find an AED, this research proposes to send the location of nearby AED along with the emergency notification.

Overall, this research aims to overcome the challenges mentioned above by designing an advanced responder network system called “Smart UseR Filter” or SURF. The authors made a previous attempt by prioritizing the factors that affect responders’ selection using Integer Linear Programming (ILP) [21]. However, the previous approach was not scalable. Therefore, a new approach has been proposed in the paper to solve the problem. The main highlights of this research are:

- Determining factors that can affect the travelling of a user to the emergency.
- Design a new algorithm for the RNS to identify users who can reach the emergency in time.
- Propose an algorithm to perform the matching between the users and the AEDs.

During a cardiac arrest, the first responder to the patient calls the emergency services requesting medical assistance. The emergency dispatcher collects the required information such as the address of the incident, patient’s symptoms and more. Based on the collected information, the nearest medical services team is dispatched to assist the patient. If the RNS is available to the emergency dispatcher, it is activated. Upon activation, the RNS notifies all the users in the 500-m radius of the emergency to assist the patient [13–15]. After receiving the emergency notification, the users find a nearby AED and carry it to the emergency. For finding a nearby AED, the user relies on either their knowledge about nearby AED locations or mobile applications, which may take away a few crucial minutes.

The method used by RNS to select users for notification is inefficient because the RNS assumes that all the users in the 500-m radius can attend the emergency, which is not always true. Another drawback of RNS is that it does not provide the location of nearby AED to the user. If RNS also provides near AED location, then the user’s time is to find an nearby AED could be saved.

**Fig. 2:**
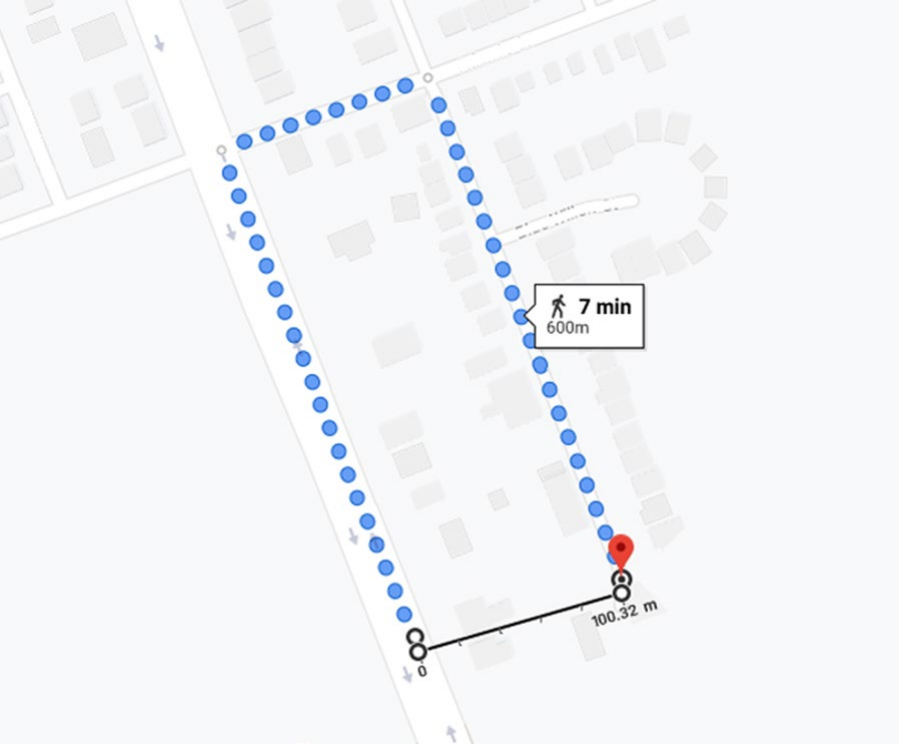
Actual travel distance versus a straight line distance between two points

The method of selecting users in a given radius from the emergency is not correct, as it is based on the assumption that any user in the radius can reach the emergency in time. This assumption is incorrect as the radius distance is not always equal to the travel distance required to reach the destination. For example, in Fig. 2, the linear distance between the two points is 100 m, but the travel distance is 600 m. In this scenario, the actual travel distance is greater than the system threshold of 500-m; however, due to the radial distance, the current system will generate an alert for this user.

The main motive behind the notification is to assist the patient at the earliest time. Therefore, the focus should be on the user’s time to reach the emergency and not on the distance from the emergency location. Distance is one factor that can help determine the time required by a user to reach an emergency. The mode of travelling (walk, bike, car), the speed of travelling (walking, running), traffic (when travelling by car) are some of the factors that also affect the time required to reach an emergency. All this information may not be available to calculate the precise travel time of a user. However, a close estimation of travel time can be determined based on the travel distance and the user’s walking speed. Furthermore, the estimated travel time can be used to identify and notify users who can reach the emergency in time.

In SURF, each user’s travel time to reach an emergency is calculated and used to notify relevant responders. The travel time is calculated based on the user’s travel distance to reach the emergency while carrying an AED on the way and their walking speed. The travel distance can be determined using publicly available maps (such as Google Maps [22]). The user’s smartphone can be used to calculate the walking speed. The smartphones use GPS and accelerometer to determine the walking speed of the user [23, 24].

**Fig. 3:**
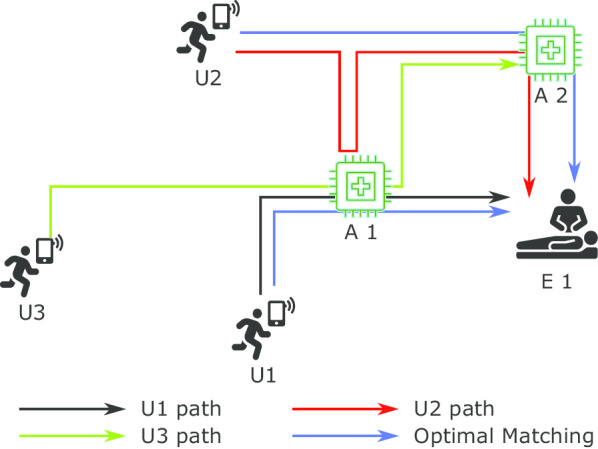
Multiple users attempting to obtain a specific AED while other AEDs are available, due to lack of knowledge

Taking a minute to find the AED on the way can reduce the survival chances of a patient by 10% [2–6]. Generally, the number of AEDs is less than the number of responders available to assist during an emergency. Thus, many users intend to carry a particular AED, but only the first user arriving at the AED location can get it. Other responders who arrive after the first user find the AED missing, and then they try to find another nearby. This situation causes delays in the arrival of users. Figure 3, demonstrates the problem. In this problem, RNS notifies users “U1”, “U2”, and “U3” to assist in the emergency “E1”. It is assumed that the users are aware of the AED (“A1” and “A2”) and their location. In this situation, all three users might think that the AED “A1” is on their way to the emergency. They all aim to carry it to the emergency. However, the user “U1” reaches the AED “A1” first and carries it to the emergency. The other two users, “U2” and “U3”, reach the AED “A1” location and finds that the AED is missing; then, they travel to AED “A2”. “U2” reaches “A2” and carries it to the emergency, but “U3” arriving after “U2” finds that the AED is missing again. In this example, “U2” has to travel twice before reaching the emergency, and “U3” travels to two AED locations. However, it is still unable to find an AED.

In the above example, the optimal answer could be that the “U1” carries “A1” to the emergency, “U2” carries “A2”, and “U3” should not be notified. If other users are not travelling, then “U3” should be notified to assist the emergency assigning the AED of the non-travelling user. SURF solves this problem by uniquely matching users with AEDs to assist in an emergency. When notifying a user about the emergency, SURF provides them with the matched AED information. By receiving the AED information, the user saves time finding a nearby AED and saving time by not travelling to multiple AED locations as the AED is uniquely matched.

**Fig. 4:**
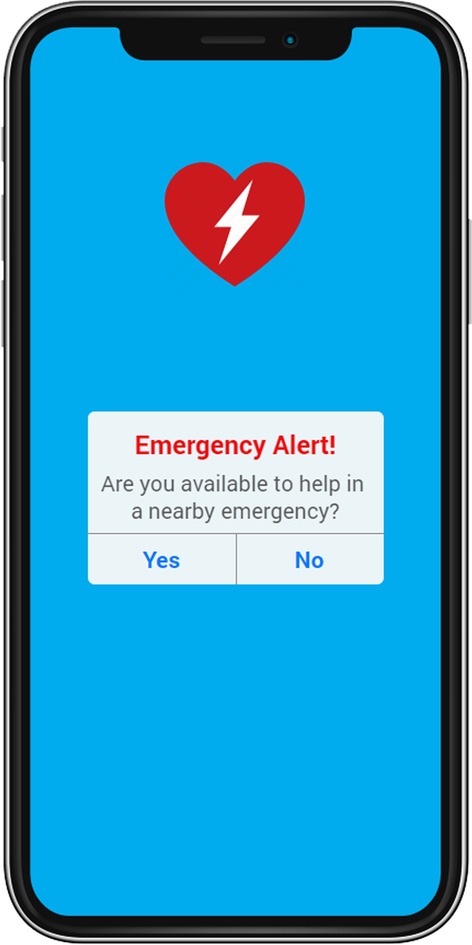
User availability confirmation screen on user’s mobile device requested by the responder network system

**Fig. 5:**
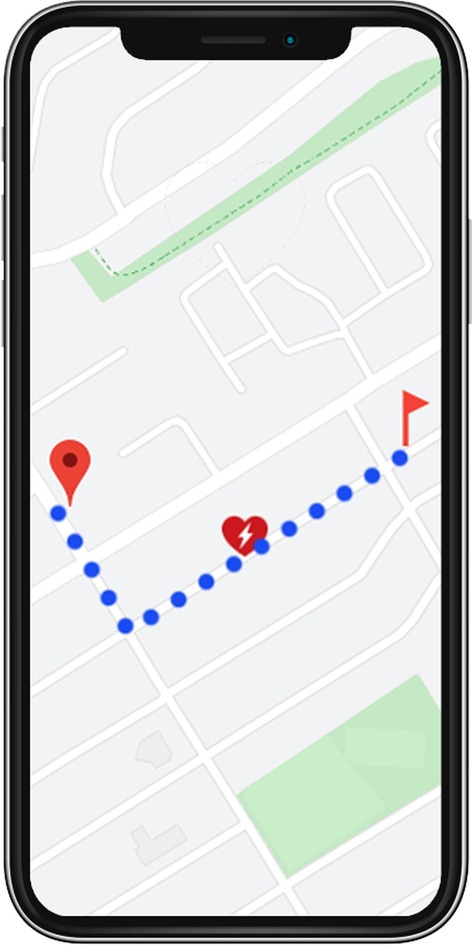
Responder network system mobile app showing the navigation path to the emergency and the matched AED on its way

The proposed system, SURF, will be deployed in the cloud and will be connected with other systems such as emergency services, AED registry and more. The user information required by SURF, such as their location, device battery level, can be collected from an existing RNS system. The AED information can be populated using an external system or an AED registry. The emergency services will send an emergency location to the SURF. Upon receiving the emergency location, the SURF performs matching between the users and the AEDs, such that the resulting pairs reach the emergency in minimum time. SURF proposes two changes to the existing emergency workflow. First, the RNS should confirm if the user is available to assist in the emergency or not, a sample shown in Fig. 4. This confirmation is required to exclude the users who are not able to assist in the emergency. Second, the RNS should send the AED information along with the emergency location and show it on the map, as shown in Fig. 5.

The objective of RNS is to provide early assistance to the patient by asking nearby users to assist the patient before emergency services arrive. There could be many nearby users (especially in densely populated cities) that will be notified to assist the patient. Therefore, RNS should notify only the users that can reach the emergency at the earliest. One way to determining these users is by identifying the time required by the users to reach the emergency. The user’s travelling time can be calculated from the distance to be travelled and the user’s walking speed. The travel distance is the distance between the user and the emergency location while carrying an AED on the way. The walking speed of the user is collected from the user’s smartphone health application.

Another factor to be considered when matching users is that the user’s device should have enough battery to keep the device powered on until they reach the emergency location. This factor can be implemented by comparing how long the user’s device can be powered and the user’s travel time. The device powered on time can be calculated based on its current battery level and its current battery consumption rate.

**Fig. 6:**
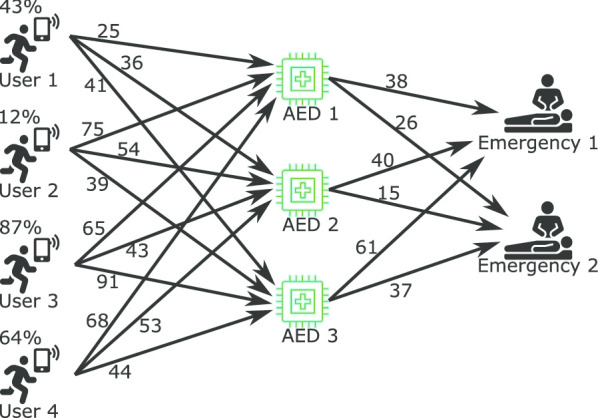
Resource matching possibilities

Figure 6, demonstrates the possible paths in which users can reach an emergency. There are 24 possible ways (4 Users * 3 AEDs * 2 Emergencies) in which the users can reach an emergency while carrying an AED. The SURF solves this constraint matching problem - matching users, AEDs, and emergencies while keeping the travel time minimum. The following constraints are added to avoid duplicate or invalid matching:

- *A user should be matched only once in a solution set* This constraint is required to avoid multiple matching of a user in the result set. For example, a user may be the nearest user for two emergencies. Then the algorithm will match the user to both emergencies. However, the user can assist only one emergency at a time.
- *An AED should be matched only once in a solution set* This constraint is required to avoid multiple matching of an AED in the result set. It is required because only a user can carry the AED at a time. Also, an AED can be used to treat a patient at a time.
- *The user’s device should have a minimum battery level* This constraint restricts the selection if the selected user’s device does not have enough battery level to keep the device powered until the user reaches the matched emergency location carrying the matched AED.

## Methods

This research focuses on matching users and AEDs with emergencies. The matching is performed based on constraints which allows only those users who can reach the emergency while carrying an AED on the way and allows matching of functional AEDs only. For performing matching, the following approaches are used in this research: Bipartite Matching (BM) and Integer Linear Programming (ILP). BM specializes in performing matching between two groups, and it has been used in industrial applications to solve matching problems [25, 26]. The application of this approach to create matches (between the users, AEDs and emergencies) is explained in “Bipartite matching” section.

The processing time the BM approach took was significantly longer for implementing the solution in a real-life situation. The causes for longer processing time were (a) a large number of variables to be matched, (b) addition of dummy variable. If the count of variables in the two matching groups is not equal, then the algorithm adds dummy variables to build a square matrix. The algorithm checks all the possible combinations among the two groups when performing matching. The addition of dummy variables increases the number of possible combinations increases, therefore increasing processing time. Thus, the ILP technique was experimented with to solve the matching problem faster.

In ILP, the matching problem is converted into a mathematical integer linear programming problem and then solved using a tool called the Gurobi Solver (Education License) [27]. ILP is being used in industries to solve the optimization and allocation of resources problems. For instance, Li et al. used ILP to optimize resource allocation for computing, and Kim et al. used ILP for scheduling power supply to minimize peak power [28, 29]. The matching problem (matching the users, AEDs and emergency) is converted into an integer linear problem and then solved using the Gurobi Solver. The matches made using ILP were similar to the matches generated using the BM approach. Also, the matches were found in significantly less time than the BM approach. However, the ILP processing time was still longer for implementation in real-life situations.

For reducing the processing time further, a new approach based on ILP is proposed called “Preprocessing with ILP”. In this new approach, the problem is solved in parts. The problem is sliced based on the travel time, allowing the matching of only those users and AEDs, which are within the sliced travel time. The problems are solved in the ascending order of their sliced travel time. Before solving each problem, a preprocessing is performed to exclude users and AEDs that have been previously matched or are outside the sliced travel time limit. With the new approach, the matching was performed faster than ILP and could be implemented in real-life situations. The technique is explained in “Pre-processing with ILP” section.

### Variables

The following are the definitions of the variables used in different approaches.

- $i = 1,2,\dotsc ,n$;    set of users
- $j = 1,2,\dotsc ,m$;   set of AEDs
- $k = 1,2,\dotsc ,o$;    set of emergencies
- $t_{ijk}$ is the time taken by user *i* to reach emergency *k* enroute to AED *j*
- $ub_{i}$ is the battery level of user *i*’s mobile device
- $ubc_{i}$ is the battery consumption rate of user *i*’s mobile device per second
- $e_{ijk}$ is the edge weight (i.e., distance) between user *i*, AED *j* and emergency *k*
- *MaxEdgeWeight* is the MAX($t_{ijk}$) + 1; $\forall i \in n ; \forall j \in m; \forall k \in o$

### Bipartite matching

In the RNS matching problem, there are three groups (users, AEDs and the emergencies) that need to be matched. However, the BM supports matching between two groups. Thus, the RNS matching problem needs to be converted from a three-dimensional to a two-dimensional problem. This conversion is performed by combining the AEDs and the emergencies into one group, as shown in step 1 of pseudocode 1 (all pseudocodes are listed in the “Appendix” section). The result of this conversion is a two-dimensional matrix as shown in Table 1. The rows and columns of the table represent two groups, and the values in the table represent edge weight(travel time) between them. The table does not have an equal number of rows and columns required to perform matching using BM. Therefore, a dummy row is required to make them equal. The edge weight for the dummy row should be distinct such that it can be easily filtered out. Thus, the edge weight for the dummy row is set to the *MaxEdgeWeight*. The *MaxEdgeWeight* is set to the highest number possible in the matrix, making it easier to identify and filter out the dummy data. The *MaxEdgeWeight* is calculated by adding one to the largest edge weight between the two groups, as shown in pseudocode 1 step 3.

**Table 1:** Matrix between users and combination of AEDs and emergencies showing Travel time (in seconds)

	${ae}_{11}$	${ae}_{12}$	$ae_{21}$	$ae_{22}$
$u_1$	12	457	68	87
$u_2$	478	57	95	35
$u_3$	75	69	41	74

Users : [$$u_1$$, $$u_2$$, $$u_3$$]

AEDs : [$$a_1$$, $$a_2$$]

Emergencies: [$$e_1$$, $$e_2$$]

Section list all the variables required for this algorithm. The preprocessing of data is performed to optimize processing. The preprocessing includes removing all the users whose mobile device does not have enough battery to keep the device powered until the user reaches the emergency, as shown in pseudocode 1 step 4. This preprocessing reduces the number of users to be matched, thus reducing processing time. The preprocessed data is then solved using the Hungarian matching algorithm. After obtaining the matching results, post-processing is performed to filter out the results. In post-processing, the following matchings are excluded: first, matches having duplicates of a user or a pair of AED and emergency by keeping the match having a lower travel time, as shown in pseudocode 1 step 6. Second, excluding matches having the travel time equal to the *MaxEdgeWeight*, as shown in pseudocode 1 step 7. Third, removing the duplicate of a user or pair of AED and emergency from the already selected matches in *M* by keeping the match having a lower travel time, shown in pseudocode 1 step 8. The matches left are added to the final result set *M*. The problem is solved again until no new acceptable matches are found. The results found are then filtered to remove pairs that have travel time equal to the *MaxEdgeWeight*, shown in pseudocode 1 step 10.

The matches generated using this approach are as expected, but the processing time required to performing matching is significantly high for implementation in real-life situations. The processing timings are discussed in detail in Section Experiment.

### Integer linear programming

For using ILP, the RNS matching problem needs to be converted into an integer linear mathematical problem. The converted problem is then solved using a solver. In this research, Gurobi Solver (Education License) is used to solve the problem [27].

The ILP result consists of either a zero or one against the decision variables, where one represents that the variable is selected for the result. When converting the RNS matching problem to an ILP problem, the decision variables are all the possible paths between the users, AEDs and emergencies, Eq. 1. If the result for a decision variable is one, then the corresponding user, AED and emergency are matched, otherwise not. For avoiding duplicate matching of users or AEDs, constraints are added, as shown in pseudocode 2 step 3 and 4. Another constraint is added to avoid creating matches in which the user’s device does not have enough charge left to keep the device powered on until the user reaches the associated the emergency location, equation in pseudocode 2 step 5.

#### Decision variables

1 $$\begin{aligned}&x_{ijk} = {\left\{ \begin{array}{ll} 1 &{} \text {user }i \text { is paired with AED }j \text { and emergency }k \\ 0 &{} \text {otherwise} \end{array}\right. }\nonumber \\&\forall i \in n ; \forall j \in m; \forall k \in o \end{aligned}$$

The objective of solving the RNS matching problem is to find the matches that can reach the emergency at the earliest while satisfying the constraints mentioned above. When solving the RNS matching problem using ILP, if the objective is set to find the matches keeping the travel time is minimum, then the output will be zero matches. It is because the travel time will be minimum when no user travels to the emergency, which is not the expected result. Therefore, the RNS problem is converted such that the objective is to maximize the travel time, as shown in pseudocode 2 step 6. This conversion requires the travel time to be inversed by subtracting the travel time from a large number, as shown in pseudocode 2 step 2. The large number is the maximum of all travel times among all possible matches, as shown in pseudocode 2 step 1.

The above-defined problem is solved using the Gurobi solver. The matches found using this approach are similar to the matches found using the BM approach in “Bipartite matching” section. Also, the processing time decreased significantly compared to the BM approach. However, the processing time is still long to implement in real-life situations. One of the reasons for longer processing time is the large input dataset, which adds a large number of variables and constraints to the problem. Thus, another approach is proposed to reduce the processing time by splitting the RNS matching problem into smaller problems.

### Pre-processing with ILP

In this approach, the ILP method is modified to reduce the processing time even further. The modification includes splitting the RNS matching problem into small problems, such that each smaller problem uses only a part of the dataset. Another modification is to use the output from the smaller problem to solve the larger problem quickly.

The RNS matching problem is split based on the travel time. The split is performed using the variables *iterationTravelTime* and *travelTimeInterval*, shown in step 3 and 4 of pseudocode 3 respectively. The *travelTimeInterval* variable is used to determine the travel time to be considered for the smallest problem. The *iterationTravelTime* variable stores the maximum travel time to be considered for each problem, and it increases by the *travelTimeInterval* after each problem is solved. When solving the problem in parts, duplicate matches will be generated as the dataset contains the previous iteration dataset. There are two ways to overcome this situation either by using constraints or pre-filtering data. Adding constraints means adding the variables again and computing more; thus, pre-filtering is chosen. Before each iteration, the pre-filtering of data is performed to exclude all paths that contain previously matched users or AEDs, as shown in pseudocode 2, step 6.

Another way to reduce the processing time is by converting the battery constraint (pseudocode 2, step 5) into a data filtering step, as showing in pseudocode 2 step 6. This conversion excludes the paths to be processed and reducing the number of constraints required.

An example of the new approach in real-life, if the emergency dispatcher wants to notify all the users that can reach the emergency in 1000 s and setting the *travelTimeInterval* to 100 s. The problem is split into iterations of 100 s (100, 200,...900, 1000). The smallest problem of 100 s is solved first. In this iteration of the problem, only the paths that have the travel time of fewer than 100 s and the paths in which the user’s battery level is higher than required. After solving the problem, the *iterationTravelTime* is increased by *travelTimeInterval* (100 s), making it to be 200 s. In the second iteration, the data is filtered by excluding all the paths that have the travel time of more than 200 s (*iterationTravelTime*). Also, excluding all the paths in which either the user or the AED is matched previously, and excluding paths where the user’s device battery level is less than the required battery level. The filtered data is used to find new matches. The iterative processing continues until the *iterationTravelTime* reaches 1000 s, and no more matching is possible. With this approach, the problem is solved quickly as each iteration is provided with a smaller dataset.

These matches generated using this approach are similar to the other two approaches (“Bipartite Matching” and “Integer linear programming”). Also, the processing time was significantly less than the other two approaches making it suitable for implementing in real-life situations. Details on processing times are explained in Section Experiment.

## Results

The proposed and the existing system can be evaluated by comparing the time taken by the notified users to reach the emergency. Also, comparing if the user chooses to carry the nearest AED, or comparing if multiple users tried to access a particular AED. The authors attempted to obtain the required data through the Institute for Clinical Evaluative Sciences (ICES) [30], hospitals and ambulance services in Canada. Unfortunately, there is no such data available to perform the comparison. Thus for the experimentation, synthetic data is generated that covers all the scenarios possible in the real world. Such as rural and urban scenarios, multiple emergencies reported at the same time, an equal number of users and AEDs available for matching.

Experiments were performed using all three approaches with a dataset of 36 test cases. In the real world, the proposed algorithm will be running on a cloud server with higher computing power than the laptop used for experimentation in this paper. The users are supposed to have mobile applications installed on their smartphones that will receive the proposed system’s notification. The experiments were executed on a laptop having an Intel i7 processor (8 core), 40 GB memory and 1TB Solid State Drive, which will eventually be replaced by the cloud-based system. The test cases represent emergency locations from a low to a highly dense populated emergency location. In the test cases, users vary between 2 and 1750, and AEDs vary between 1 to 250. In densely populated cities, it is possible to have more than one emergency occurring nearby. Thus, multiple emergencies were added to the test data to cover such scenarios. A comparison is made between the results from all three approaches to compare the processing time and the total number of unique emergencies covered, shown in Table 2. It presents the processing time to solve each test case by the three approaches. The Preprocessed ILP approach took significantly lower processing time when compared with the other two approaches. With the increasing number of variables (users, AEDs and emergencies), the processing time of Preprocessed ILP decreased when compared with other approaches. The most comprehensive test case in this experiment is test case 36 with 1750 users, 250 AEDs, and five emergencies. The Hungarian approach took 11,823.76 s to solve the problem, while the ILP approach took 143.14 s, and the Preprocessed ILP approach took 6.04 s. The Hungarian approach took such a long processing time because of the large number of variables to be matched. In test case 36, the Hungarian approach had to match between 1750 users and 1250 (AEDs $*$ emergencies). In contrast, in ILP approach, the number of variables was 2,187,500 (total matches possible) and 5250 constraints, but the problem solved faster due to the linear programming approach. In the Preprocessed ILP approach with *iterationTravelTime* equal to 100 s, there were 297,952 variables and 1,844 constraints in the first iteration, which is approximately 14% and 35% of the ILP approach, respectively. In the following rounds, the number of variables and constraints were reduced by more than 60%, making the problem solve faster.

**Table 2:** Experiment data with results using all three different approaches

Test case	Users	AEDs	Emergencies	Processing time (s)			Unique emergencies covered		
				Hungarian	ILP	Preprocessed ILP	Hungarian	ILP	Preprocessed ILP
1	50	2	1	0.01	0.06	0.03	1	1	1
2	100	3	1	0.05	0.08	0.01	1	1	1
3	150	4	1	0.10	0.09	0.02	1	1	1
4	200	5	1	0.24	0.07	0.02	1	1	1
5	250	6	1	0.42	0.09	0.02	1	1	1
6	300	7	1	0.74	0.16	0.03	1	1	1
7	350	8	1	1.06	0.19	0.05	1	1	1
8	400	9	1	1.53	0.24	0.05	1	1	1
9	450	10	1	2.20	0.21	0.04	1	1	1
10	500	10	1	2.55	0.23	0.05	1	1	1
11	550	10	1	2.97	0.24	0.04	1	1	1
12	600	20	1	6.95	0.48	0.06	1	1	1
13	650	30	1	13.01	0.76	0.12	1	1	1
14	700	40	1	19.66	1.21	0.16	1	1	1
15	750	50	1	34.10	1.69	0.31	1	1	1
16	800	60	2	105.18	5.76	0.35	2	2	2
17	850	70	2	146.26	7.10	0.45	2	2	2
18	900	80	2	183.78	8.84	0.54	2	2	2
19	950	90	2	222.70	9.53	0.63	2	2	2
20	1000	100	2	251.78	11.68	0.78	2	2	2
21	1050	110	3	571.15	20.10	1.08	3	3	3
22	1100	120	3	689.33	23.73	1.20	3	3	3
23	1150	130	3	843.60	26.63	1.46	3	3	3
24	1200	140	3	1010.60	32.79	1.42	3	3	3
25	1250	150	3	1112.83	41.13	1.68	3	3	3
26	1300	160	4	2178.66	61.08	2.06	4	4	4
27	1350	170	4	2359.92	67.09	2.40	4	4	4
28	1400	180	4	2969.93	75.04	3.08	4	4	4
29	1450	190	4	3329.03	80.15	2.94	4	4	4
30	1500	200	4	3671.05	83.53	3.23	4	4	4
31	1550	210	5	7682.36	120.98	4.00	5	5	5
32	1600	220	5	8442.53	128.44	4.74	5	5	5
33	1650	230	5	9353.51	130.15	4.95	5	5	5
34	1700	240	5	11,387.03	139.14	4.73	5	5	5
35	1750	250	5	11,823.76	143.14	6.04	5	5	5
$36^{{\mathrm{a}}}$	4	4	2	0.001	0.005	0.003	1	1	2

${}^{{\mathrm{a}}}$A special case to show the greedy matching performed by Hungarian and ILP approaches

In all three approaches, the resulting matches were similar. The results can also be compared against the count of matches found and the total match time. The counts of matches are equal in all test cases among the three approaches. The total match time is the sum of the travel time for all the selected matches to reach their matched emergency while carrying the matched AED. The total match time for Preprocessed ILP is either equal or less than the other two approaches. This comparison confirms that the Preprocessed ILP matching process is better than the other two approaches.

In the Preprocessed ILP approach, the *travelTimeInterval* variable can be modified depending on the city’s population density. A higher *travelTimeInterval* is better if the city is less populated, and lower *travelTimeInterval* for dense cities. The appropriate *travelTimeInterval* value also can help in decreasing the processing time ever further. The processing time can also be reduced by limiting the number of matches to be selected for an emergency. When the required number of matches are found for the emergency, the process is stopped between outputting the matches found.

**Fig. 7:**
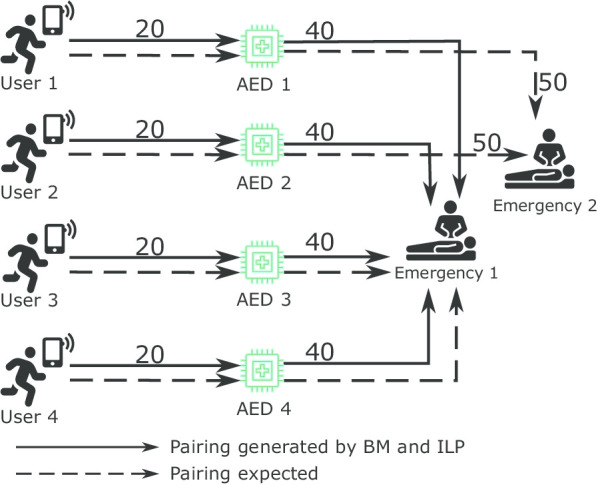
Figure shows the greedy approach for match generation considering only to reduce time to reach the emergency, which in this case, leaves the Emergency 2 unattended

During the experimentation, a new problem was discovered that is the greedy approach of matching. In all three approaches, the objective was to perform matching such that the users reach the emergency at the earliest. However, in some cases, the emergencies remained unattended due to the greedy matching. The column “Unique Emergencies Covered” in Table 2 represents the number of different emergencies covered in matches found. Test Case 36 is a clear example of the greedy matching approach, in which all three approaches found matches attending a particular emergency only, and the other emergency was not attended, shown in Fig. 7. All three approaches found the following matches “U1-A1-E1”, “U2-A2-E1”, “U3-A3-E1”, and “U4-A4-E1” because the travel time was least among other possible matches. However, the excepted matches for this problem are: “U1-A1-E2”, “U2-A2-E2”, “U3-A3-E1”, and “U4-A4-E1”, such that both the emergencies are attended.

The solution to the greedy approach of matching is enforcing an equal distribution of users and AEDs among emergencies. This enforcement is applied by adding a secondary objective to the Preprocessed ILP approach. For implementing this objective, the count of matches per emergency is required. It is calculated by counting the number of matches found for each emergency, as shown in Eq. 2. The next step is to identify the minimum number of matches found among all emergencies, as shown in Eq. 3. The previous step’s outputted value is set to be maximized for the secondary objective, shown in Eq. 4.

2 $$\begin{aligned}&f_{minEmergency}(k) = \sum _{i=1}^{n} \sum _{j=1}^{m} \big ( x_{ijk} \big ); \forall i \in n ; \forall j \in m \end{aligned}$$

3 $$\begin{aligned}&MinPair = Min(f_{minEmergency}(k));\forall k \in o \end{aligned}$$

4 $$\begin{aligned}&Max(MinPair) \end{aligned}$$

All test cases were executed again with the secondary objective in the Preprocessed ILP approach. Table 3 presents the comparison of before and after the addition of the secondary objective to the Preprocessed ILP approach. The processing time increased after adding the secondary objective to the Preprocessed ILP approach. The increase in the processing time is not significant, except in the test case 36. The reason for the increased processing time is that a large number of variables need to be optimized for the secondary objective. The difference in the processing time can be reduced if the *travelTimeInterval* is set to a lower number. If the *travelTimeInterval* is set to 50 s, then the problem is solved in 9.08 s.

**Table 3:** A comparison between preprocessed ILP approach (PP ILP) and preprocessed ILP approach with secondary emergency objective (PP ILP E)

Test case	Processing time		Result travel time		Matches per emergency	
	PP ILP	PP ILP E	PP ILP	PP ILP E	PP ILP	PP ILP E
1	0.03	0.02	573.01	573.01	‘e1’: 2	‘e1’: 2
2	0.01	0.01	361.68	361.68	‘e1’: 3	‘e1’: 3
3	0.02	0.02	1222.13	1222.13	‘e1’: 4	‘e1’: 4
4	0.02	0.02	1482.39	1482.39	‘e1’: 5	‘e1’: 5
5	0.02	0.02	1739.23	1739.23	‘e1’: 6	‘e1’: 6
6	0.03	0.03	2112.71	2112.71	‘e1’: 7	‘e1’: 7
7	0.05	0.03	1460.81	1460.81	‘e1’: 8	‘e1’: 8
8	0.05	0.05	3762.28	3762.28	‘e1’: 9	‘e1’: 9
9	0.04	0.03	2128.04	2128.04	‘e1’: 10	‘e1’: 10
10	0.05	0.05	2778.93	2778.93	‘e1’: 10	‘e1’: 10
11	0.04	0.04	3821.47	3821.47	‘e1’: 10	‘e1’: 10
12	0.06	0.07	4180.45	4180.45	‘e1’: 20	‘e1’: 20
13	0.12	0.11	9095.84	9095.84	‘e1’: 30	‘e1’: 30
14	0.16	0.16	14,195.57	14,195.57	‘e1’: 40	‘e1’: 40
15	0.31	0.27	15,062.98	15,062.98	‘e1’: 50	‘e1’: 50
16	0.35	0.42	11,795.09	11,923.86	‘e1’: 28, ‘e2’: 32	‘e1’: 30, ‘e2’: 30
17	0.45	0.54	13,755.34	13,796.98	‘e1’: 29, ‘e2’: 41	‘e1’: 32, ‘e2’: 38
18	0.54	0.71	17,409.57	17,529.67	‘e2’: 48, ‘e1’: 32	‘e2’: 45, ‘e1’: 35
19	0.63	0.80	15,936.44	16,034.09	‘e2’: 50, ‘e1’: 40	‘e2’: 48, ‘e1’: 42
20	0.78	1.01	17,545.64	17,553.02	‘e2’: 49, ‘e1’: 51	‘e2’: 50, ‘e1’: 50
21	1.08	1.26	18,073.04	18,225.54	‘e3’: 34, ‘e2’: 29, ‘e1’: 47	‘e3’: 34, ‘e2’: 31, ‘e1’: 45
22	1.20	1.39	17,307.57	17,399.31	‘e3’: 43, ‘e2’: 35, ‘e1’: 42	‘e3’: 41, ‘e2’: 39, ‘e1’: 40
23	1.46	1.64	21,938.17	22,138.55	‘e3’: 37, ‘e2’: 53, ‘e1’: 40	‘e3’: 38, ‘e2’: 49, ‘e1’: 43
24	1.42	1.66	19,297.70	19,541.02	‘e2’: 53, ‘e3’: 44, ‘e1’: 43	‘e2’: 49, ‘e3’: 46, ‘e1’: 45
25	1.68	1.86	22,488.18	22,830.32	‘e2’: 44, ‘e1’: 58, ‘e3’: 48	‘e2’: 49, ‘e1’: 53, ‘e3’: 48
26	2.06	2.40	20,339.94	20,810.00	‘e4’: 44, ‘e3’: 41, ‘e2’: 32, ‘e1’: 43	‘e2’: 38, ‘e3’: 39, ‘e1’: 42, ‘e4’: 41
27	2.40	3.75	21,888.44	22,425.71	‘e1’: 39, ‘e2’: 42, ‘e4’: 45, ‘e3’: 44	‘e1’: 40, ‘e2’: 44, ‘e4’: 44, ‘e3’: 42
28	3.08	4.26	22,012.02	22,478.28	‘e4’: 41, ‘e2’: 48, ‘e1’: 48, ‘e3’: 43	‘e4’: 48, ‘e2’: 47, ‘e1’: 45, ‘e3’: 40
29	2.94	3.44	22,690.02	22,920.88	‘e4’: 42, ‘e1’: 53, ‘e2’: 44, ‘e3’: 51	‘e4’: 49, ‘e1’: 50, ‘e2’: 46, ‘e3’: 45
30	3.23	3.83	24,983.04	25,384.24	‘e4’: 49, ‘e2’: 61, ‘e1’: 41, ‘e3’: 49	‘e4’: 50, ‘e2’: 50, ‘e1’: 48, ‘e3’: 52
31	4.00	4.88	20,898.51	21,377.67	‘e3’: 36, ‘e4’: 52, ‘e1’: 36, ‘e2’: 41, ‘e5’: 45	‘e3’: 42, ‘e4’: 42, ‘e1’: 40, ‘e2’: 40, ‘e5’: 46
32	4.74	5.94	22,856.85	23,082.65	‘e3’: 40, ‘e4’: 46, ‘e5’: 45, ‘e1’: 44, ‘e2’: 45	‘e3’: 43, ‘e4’: 45, ‘e5’: 45, ‘e1’: 44, ‘e2’: 43
33	4.95	5.81	23,183.70	23,713.83	‘e2’: 37, ‘e4’: 37, ‘e1’: 52, ‘e3’: 54, ‘e5’: 50	‘e2’: 41, ‘e4’: 41, ‘e1’: 45, ‘e3’: 53, ‘e5’: 50
34	4.73	6.04	24,452.90	25,142.43	‘e3’: 38, ‘e5’: 47, ‘e2’: 51, ‘e1’: 47, ‘e4’: 57	‘e3’: 46, ‘e5’: 48, ‘e2’: 50, ‘e1’: 48, ‘e4’: 48
35	6.04	13.08	26,145.18	26,643.97	‘e1’: 49, ‘e3’: 51, ‘e2’: 44, ‘e5’: 52, ‘e4’: 54	‘e4’: 49, ‘e3’: 51, ‘e1’: 49, ‘e2’: 50, ‘e5’: 51
$36^{{\mathrm{a}}}$	0.00	0.01	240.00	260.00	‘e1’: 4	‘e2’: 2, ‘e1’: 2

The processing time and match total time measured in seconds

${}^{{\mathrm{a}}}$A special case to show the greedy matching performed by Hungarian and ILP approaches

The results were also compared against the result travel time. The result travel time is calculated by adding the travel time of all the matches found in the individual test case. The result travel time is slightly increased after adding the secondary objective. An increase in time is expected when changing from the greedy approach of matching to an equal distribution. Another comparison is performed on the number of matches found per emergency to verify the equal matches among emergencies.

Overall, the secondary objective performed as expected by equally distributing the users and the AEDs among emergencies. Test case 36 clearly shows the difference between the two approaches. The Preprocessed ILP performed the greedy approach of matching; therefore, it assigned all users and AEDS to emergency “e1”, and the result travel time totalled 240 s. However, the Preprocessed ILP having the secondary objective distributed the users and the AEDs equally among the two emergencies with the result travel time totalled to 260 s.

## Discussion

It is crucial to provide resuscitation to the patient during a cardiac arrest emergency by performing CPR and applying AED immediately. The emergency services are not always able to reach the patient immediately. Thus, an RNS was created to identify and notify registered users nearby the emergency location to assist the patient before the arrival of emergency services. After implementing the RNS, the patient’s survival rate increased, showcasing the effectiveness of this system. The existing RNS systems have drawbacks, if resolved, can reduce the time for resuscitation. The proposed algorithm solves some of these drawbacks. The proposed algorithm notifies only the users that can reach the emergency in time and provides them with information about the nearest AED available for use. The system can also handle multiple emergencies at the same time.

Future work will entail collecting real-world data through collaboration with an existing RNS company. The collaboration work will include collecting the information required to test the proposed system and comparing the proposed system with existing RNS systems. Some constraints in the proposed system can be relaxed to include more users or AEDs. Such as, it is required that the user’s device battery last until the end of the emergency. However, this constraint can be relaxed by including the users whose device battery can keep the device powered up until they are a few meters away from the emergency location. Other factors that can be considered while performing matching are the battery level of the AEDs, different modes of travelling, user’s CPR certification status, and many more. The proposed system can also be enhanced to track the movement of the notified user, and if the user is not moving as expected, then the system can match the AED associated with another user to maximize the possibility of someone reaching the emergency.

In future, the proposed system will be integrated with an indoor navigation system that will help the users to navigate and find AEDs within complicated premises, like an office building with 20 floors and having thousands of meters of floor area. This integration will help in measuring the distance between the user and the AED more precisely.

Another implementation of the proposed system can be to dispatch different emergency units (ambulance, fire, police) based on the type of emergency reported. For example: in case of theft, nearby police units can be dispatched. In case of a car accident, nearby police and ambulance can be dispatched.

## Conclusion

This paper proposes a new algorithm, SURF, to solve AED’s matching problem with the user(s). in this paper. SURF determines the users to be alerted for an emergency based on the travel time to reach the emergency. The information about the users, AEDs, and the emergency location is used to generate matches. The matching information, which includes the emergency and the AED locations, is then shared with the associated user. The AED location information saves the time spent by the user to find a nearby AED. During matching, a unique AED is assigned to a user, which helps avoid situations where multiple users get a particular AED. The results show that the proposed algorithm’s matching is faster than the existing matching techniques (BM, ILP), thus making it possible to be implemented in the real world.

## Data Availability

The datasets used and/or analysed during the current study are available from the corresponding author on reasonable request.

## Abbreviations

SURF: Smart UseR Filter;
OHCA: Out-of-Hospital Cardiac Arrest;
EMS: Emergency medical services;
RNS: Responder network system;
AED: Automated External Defibrillator;
ILP: Integer Linear Programming;
IoT: Internet of things;
SCA: Sudden Cardiac Arrest;
CPR: Cardiopulmonary Resuscitation;
BM: Bipartite Matching;
